# Epidemiology, Clinical Characteristics, and Hospitalization Patterns in Children Aged ≤72 Months with Traumatic Head Injury: A 16-Year Single-Center Study

**DOI:** 10.3390/children13101382

**Published:** 2026-10-12

**Authors:** Miloš Pajić, Jelena Antić, Goran Rakić, Ivana Fratrić, Branislava Brestovački Svitlica

**Affiliations:** 1 Faculty of Medicine, University of Novi Sad, 21000 Novi Sad, Serbiajelena.antic@mf.uns.ac.rs (J.A.); branislava.brestovacki@mf.uns.ac.rs (B.B.S.); 2 Institute for Children and Youth Healthcare of Vojvodina, 21101 Novi Sad, Serbia

**Keywords:** traumatic head injury, pediatrics, hospitalization, falls, injury severity

## Abstract

**Highlights:**

**What are the main findings?**

• Traumatic head injuries in young children are predominantly mild and most commonly result from falls, particularly in the home environment.
• Severe clinical courses and the need for surgical treatment are relatively uncommon in this age group.

**What are the implications of the main findings?**

• Over the 16-year study period at a Serbian tertiary pediatric center, annual head-injury examinations declined while the proportion hospitalized increased markedly and nonlinearly. This divergence provides a local service-use perspective beyond descriptions of injury mechanisms.
• Among hospitalized children, infancy, ambulance transport, interfacility transfer, and selected non-fall injury mechanisms were independently associated with a severe clinical course. These associations describe the admitted cohort and do not constitute a validated admission rule.

**Abstract:**

**Purpose**: To characterize hospitalized children aged ≤72 months with traumatic head injury at a Serbian tertiary pediatric center, evaluate factors associated with severe clinical course as the primary patient-level outcome, and describe temporal patterns in hospitalization among head-injury presentations. **Methods**: This retrospective single-center study covered 2010–2025. Patient-level data were available for hospitalized children, while annual aggregate counts described head-injury presentations. Severe clinical course was defined as recorded GCS ≤ 8 on admission, surgical intensive care unit admission, and/or surgical intervention. Multivariable logistic regression evaluated associated factors, and quasi-binomial regression examined temporal changes in hospitalization proportions. **Results**: A cohort of 971 hospitalized children was analyzed. Over the study duration, a decrease in the absolute number of head injury evaluations was observed, whereas the proportion of hospitalizations increased from 11.6% to 58.0%. The predominant mechanism of injury was domestic falls from heights. Most children (98.2%) were managed conservatively. A severe clinical course was documented in 70 children (7.2%). Factors significantly associated with higher odds of a severe clinical course included infancy (age ≤ 12 months), ambulance transport, inter-institutional transfer, and traffic-related incidents. **Conclusions**: The distinctive finding was a rising hospitalization proportion despite fewer head-injury presentations. This describes changing service use rather than establishing an increase in injury severity or identifying avoidable admissions. The findings support evaluation of local admission and observation pathways and improved clinical documentation; the causes of the temporal trend require further study.

## 1. Introduction

Unintentional injuries constitute a leading cause of morbidity and mortality among children globally, representing a critical public health priority [1]. Traumatic head injuries (THI) are among the most prevalent and clinically significant injuries in the pediatric population. Due to their incidence and the potential for enduring neurocognitive and psychosocial sequelae, they pose a substantial challenge to healthcare systems [2,3]. Epidemiological data indicate that THI is a frequent indication for emergency department visits and hospitalizations, particularly among preschool-aged children, thereby contributing significantly to the global burden of pediatric trauma [3,4,5,6,7].

Although severe intracranial injuries and the requirement for surgical intervention are relatively uncommon, mortality rates in severe THI can reach 12% [4,5]. Despite the clinical implementation of validated decision rules designed to optimize hospitalization and neuroimaging protocols, admission practices remain heterogeneous across healthcare systems, with a high proportion of admissions involving mild injuries [8]. The mechanisms of injury exhibit age-dependent variations; falls represent the most frequent cause of THI, especially in younger children, where injuries predominantly occur within the domestic environment during routine activities [4]. Children under the age of six exhibit particular vulnerability owing to their proportional head dimensions, immature motor coordination, and exploratory behaviors [2]. Although road traffic accidents occur less frequently, they are correlated with increased injury severity and a higher probability of hospitalization [5]. Considering that traumatic insults during critical phases of neurological development may result in protracted sequelae—including cognitive deficits, attentional disorders, and social maladaptation [3,9,10]—preventive strategies and early identification are imperative [11]. In Serbia, a national registry for pediatric traumatic head injury (THI) is currently unavailable. While existing reports indicate that injuries and poisonings constitute primary causes of hospitalization among preschool-aged children [12], granular hospital-based data regarding THI within this demographic remain scarce. International literature has established falls as the predominant injury mechanism in young children and identified infancy and high-energy mechanisms as indicators of heightened clinical concern [4,5,7]. Consequently, the present study provides a 16-year, single-center characterization of hospitalized children aged ≤72 months in Serbia, evaluates factors associated with severe clinical progression within this cohort, and formally analyzes the annual proportion of hospitalizations among pediatric patients presenting with head injuries. This study was not designed to identify patient-level determinants for hospitalization, as individual-level data were restricted to the hospitalized population.

## 2. Methodology

### 2.1. Study Design and Setting

A retrospective, single-center observational study was conducted between 1 January 2010 and 31 December 2025, at the Pediatric Surgery Clinic of the Institute for Children and Youth Health Care of Vojvodina, a tertiary referral pediatric institution catering to a patient population aged 0 to 18 years. As the preeminent pediatric center in the Autonomous Province of Vojvodina, the Institute provides highly specialized diagnostic and therapeutic services to a catchment area of approximately 2 million inhabitants. Patients requiring advanced clinical interventions are referred to this facility from secondary-level regional hospitals.

### 2.2. Participants

The study cohort comprised consecutive hospitalizations of pediatric patients aged ≤72 months (up to and including 6 years, excluding children older than 72 months) who, following a traumatic head injury (THI), underwent initial evaluation at the emergency department of the Children’s Surgery Clinic prior to admission. The unit of analysis was defined as the individual hospitalization episode. To mitigate multiple-measurement bias, only the primary hospitalization was included in the analysis for patients with recurrent admissions.

### 2.3. Case Identification

Cases were identified by searching the hospital information system using the primary discharge ICD-10 diagnoses S06.0 (concussion), S02.0 (fracture of vault of skull), and S00.9 (superficial injury of head, unspecified). These were the most frequently used pediatric head-injury codes at our institution and were consistently available throughout the study period, enabling a uniform retrospective case-identification strategy. These primary codes did not exclude coexisting intracranial or other cranial injuries. However, children whose primary discharge diagnosis fell outside the selected codes may have been missed; the cohort should not be interpreted as capturing the complete institutional burden of pediatric head injury. The study period began in 2010, when electronic medical records were introduced at our institution, and ICD-10 was used throughout 2010–2025.

**Figure 1:**
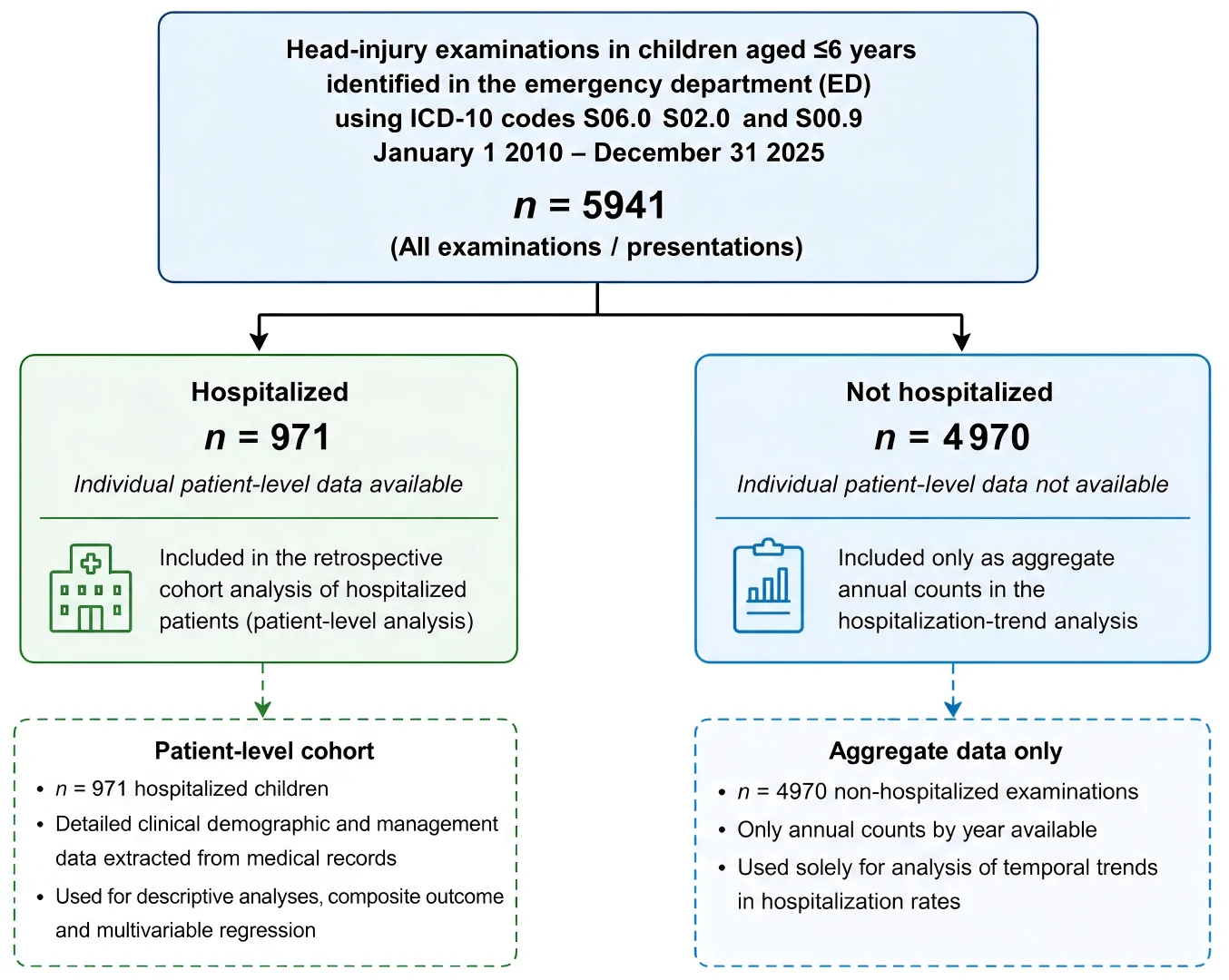
Flow diagram of study population and data availability.

The study population and the availability of patient-level versus aggregate data are summarized in Figure 1.

### 2.4. Inclusion and Exclusion Criteria

Criteria for inclusion in the study: children aged ≤72 months with specified ICD-10 diagnoses and available complete medical documentation. Exclusion criteria for the study: patients older than 72 months and cases with insufficient data to reliably classify the clinical course or outcome.

### 2.5. Data Sources and Data Collection

Data were collected retrospectively from electronic medical records and discharge sheets. One investigator performed data extraction, and another investigator conducted quality control on a random sample of approximately 20% of cases. Key variables examined included age, sex, mechanism of injury, Glasgow Coma Scale (GCS), diagnostic procedures, intensive care unit treatment, surgical interventions, and length of hospitalization. Any discrepancies identified during quality control were resolved by re-review of the source documentation. All data were anonymized before analysis.

### 2.6. Variables

The primary patient-level outcome was defined as a severe clinical course, defined for this analysis as a recorded Glasgow Coma Scale (GCS) score ≤8 upon admission, requirement for surgical intensive care unit admission, and/or surgical intervention during hospitalization. Secondary descriptive outcomes included injury mechanisms, clinical and imaging characteristics, length of stay, treatment, and recorded discharge status. Annual hospitalization proportions were examined as a separate aggregate service-use analysis. Prespecified predictors included sex, age group (≤12 months, 13–36 months, and 37–72 months), place of residence (rural or urban), mode of arrival (parental transport, ambulance transport, or inter-facility transfer), and injury mechanism (falls, direct impact/crush, traffic-related injury, or other/unknown). Clinical symptoms were categorized into five predefined domains based on medical documentation: neurological symptoms (vomiting, headache, loss of consciousness, amnesia, confusion, or drowsiness), somatic symptoms (swelling, hematoma, or pain), psychological/behavioral symptoms (agitation, irritability, or behavioral changes), unreliable data, and nonspecific symptoms. The “unreliable data” category was utilized when documentation precluded a definitive determination of the presenting symptom, whereas “nonspecific symptoms” referred to documented symptoms that did not align with the three primary clinical categories. These variables were selected based on clinical relevance, existing literature regarding pediatric traumatic brain injury, and data availability within the retrospective medical records.

### 2.7. Data Analysis

Aggregated yearly data were used to describe trends in head-injury examinations and hospitalizations, and the proportion of hospitalized children among those examined for head injury. Temporal changes in the proportion hospitalized were formally examined using quasi-binomial logistic regression fitted to the annual numbers of hospitalized and non-hospitalized children. Year was centered at 2010. A model containing a linear effect of year was compared with a model additionally containing a quadratic year term, using an F-test, to assess whether allowing for a nonlinear temporal pattern improved model fit. Annual 95% score confidence intervals for the observed hospitalization proportions were calculated without continuity correction. Patient-level data were summarized as frequencies and percentages, and hospital length of stay was described as the median, interquartile range, and range. Severe clinical course was defined as a composite outcome (recorded GCS ≤ 8, surgical intensive care unit admission, and/or surgical intervention). This approach was used due to the limited availability of GCS and the lack of a single reliable marker of severity in this retrospective cohort. A multivariable logistic regression model was fitted with sex, age group, place of residence, mode of arrival, and injury mechanism as predictors. To assess the robustness of the primary regression model to temporal adjustment and potential small-sample bias, the model was additionally fitted with calendar year as a covariate and was re-estimated using Firth penalized logistic regression. The Firth model was also repeated with calendar year included as an additional covariate. Two additional sensitivity analyses were performed to examine the robustness of the findings to alternative definitions of severe clinical course. First, the primary multivariable logistic regression was repeated using an outcome defined only by treatment in the surgical intensive care unit and/or surgical intervention, thereby excluding GCS. Second, a more restrictive analysis used surgical intervention alone as the outcome. Because surgical intervention was rare, occurring in 17 children, this model was estimated using Firth penalized logistic regression to reduce small-sample bias associated with sparse outcome data. Multicollinearity, overdispersion, and complete or quasi-complete separation were assessed for the primary multivariable logistic regression model. Model explanatory performance was summarized using Tjur’s R^2^. Discrimination was assessed using the 5-fold cross-validated area under the receiver operating characteristic curve (AUC). Calibration was assessed graphically and using the Hosmer–Lemeshow goodness-of-fit test. Results from the multivariable models are presented as adjusted odds ratios (aORs) with 95% confidence intervals and *p*-values. The significance level was set at α = 0.05. All analyses were conducted using R 4.6.0.

### 2.8. Ethics

This study was conducted in accordance with the ethical principles of the Declaration of Helsinki. The consent of the Commission for the Ethics of Examination of the Faculty of Medicine in Novi Sad (01-39/98) and the consent of the Ethics Committee of the Institute for Health Care of Children and Youth of Vojvodina (17/22) were obtained.

## 3. Results

### 3.1. Trends in Head-Injury Examinations and Hospitalizations Among Children Aged ≤72 Months

**Figure 2:**
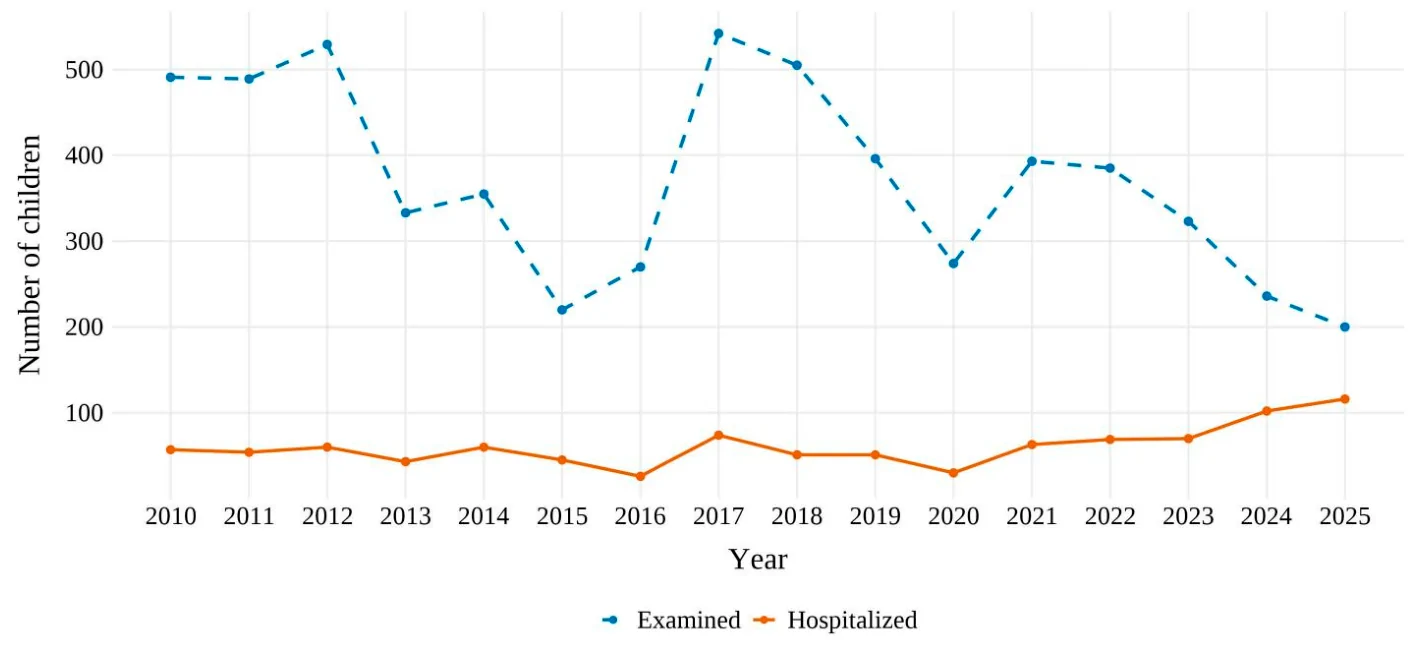
Head Injuries Among Children Aged ≤72 Months: Examinations and Hospitalizations.

**Figure 3:**
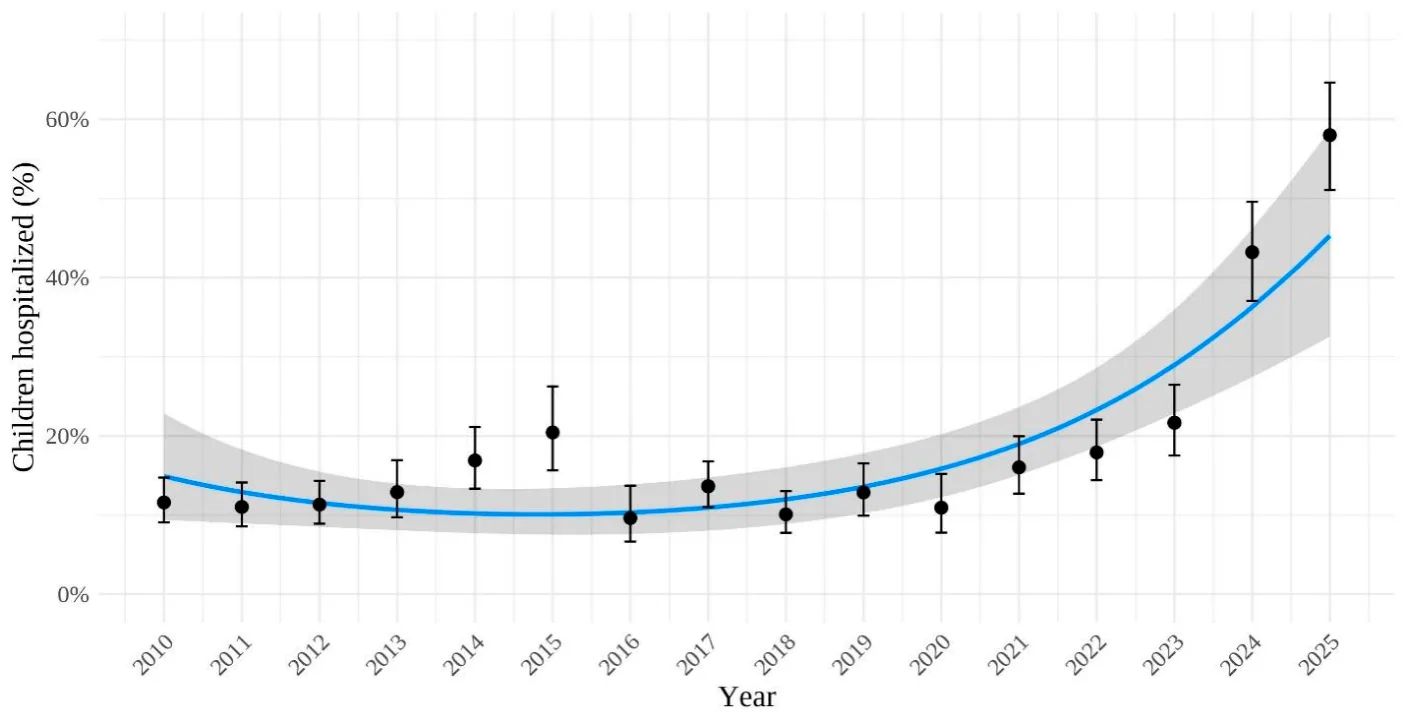
Observed and Model-Predicted Hospitalization Proportions Among Children Aged ≤72 Months Examined for Head Injury, 2010–2025. The grey shaded area represents the 95% confidence interval around the fitted temporal trend shown by the blue line.

During the 2010–2025 study period, 5941 emergency department presentations for head injury among children aged ≤72 months were recorded, of which 971 resulted in hospitalization. The aggregated data obtained for the period 2010–2025 indicates that, among children aged ≤72 months with head injury, the number of examinations decreased while the number of hospitalizations increased. Specifically, the number of examinations decreased from 491 in 2010 to 200 in 2025, whereas the number of hospitalizations increased from 57 to 116. In other words, the proportion of hospitalized children among those examined for head injury increased from 11.6% in 2010 to 58.0% in 2025. Figure 2 shows the yearly number of examined and hospitalized children aged ≤72 months with head injury. Formal analysis indicated that the temporal pattern in the proportion hospitalized was nonlinear. A quadratic model provided a significantly better fit than a model containing only a linear effect of year, F(1, 13) = 13.91, *p* = 0.003. The fitted pattern was consistent with a relatively modest change during the earlier part of the study period followed by a more pronounced increase in hospitalization proportions during the later years. This pattern is also evident in Figure 3, with the highest proportions observed in 2024 (43.2%) and 2025 (58.0%).

In Figure 3, black points represent the observed annual proportions of children hospitalized among those examined for head injury, with error bars indicating 95% confidence intervals for the observed proportions. The blue line represents the fitted hospitalization proportions from the quadratic quasi-binomial logistic regression model, and the gray shaded area represents the 95% confidence interval around the model-predicted proportions.

### 3.2. Demographic and Seasonal Characteristics of Hospitalized Children Aged ≤72 Months

Table 1 summarizes the demographic characteristics of the patient sample. Boys and girls were relatively evenly represented. Most children belonged to the oldest age group, while the youngest children were least represented. Urban residence was slightly more common than rural residence. Seasonally, injuries were most frequent in summer and fall and least frequent in winter.

### 3.3. Mechanism of Injury and Mode of Arrival

As presented in Table 2, falls from height inside the home were the most frequent mechanism of head injury, followed by falls on the same level and blows or collisions during play. The least frequent recorded causes were birth trauma and abuse, while a small proportion of injuries had an unknown cause. Most children were brought to the hospital by their parents (*n* = 701, 72.2%), followed by ambulance transport (*n* = 150, 15.4%) and transfer from another healthcare facility (*n* = 120, 12.4%).

### 3.4. Clinical Characteristics of Injury

The GCS was not documented in 90.8% of cases. Among the 89 children with a recorded GCS score, most had a score of 15, followed by scores of 13–14; moderate and severe GCS categories were rare. The components of the severe clinical course outcome overlapped partially. Overall, 66 children (6.8%) were admitted to the surgical ICU and 17 (1.8%) underwent surgical intervention, including 13 who met both criteria; therefore, 70 children (7.2%) met the composite outcome definition. Among the 70 children meeting the composite definition, 47 were admitted to the ICU without GCS ≤ 8 or surgical intervention, 6 had GCS ≤ 8 and were admitted to the ICU, 13 were both admitted to the ICU and underwent surgical intervention, and 4 underwent surgical intervention without ICU admission. No child with GCS ≤ 8 underwent surgical intervention, and no child met all three criteria. Thus, all six children with GCS ≤ 8 were already identified by ICU admission and did not contribute additional cases to the composite outcome. In 2024, there were two deaths. The cause was a traffic accident, and the children were female and under 12 months old. Table 3 presents the remaining clinical characteristics, diagnostic procedures, hospitalization characteristics, and treatment modalities.

Concussion was the most frequent diagnosis (*n* = 465, 47.9%), followed by superficial head injury (*n* = 301, 31.0%) and skull-vault fracture (*n* = 205, 21.1%). Neurological symptoms were the most commonly recorded symptom category (*n* = 678, 69.8%), followed by somatic symptoms (*n* = 195, 20.1%). Skull radiography was the most frequently used diagnostic procedure (*n* = 659, 67.9%), whereas computed tomography (CT) was less common (*n* = 197, 20.3%) and sometimes combined with it. Most children stayed in the hospital for more than one day (*n* = 565, 58.2%), were treated on the ward rather than in surgical intensive care (*n* = 905, 93.2%), and received conservative treatment (*n* = 954, 98.2%). Median hospitalization length was 2 days (IQR = 2), with a range of 1–27 days.

### 3.5. Surgical Treatment

Seventeen children (1.8% of the hospitalized cohort) underwent surgical treatment. The individual clinical characteristics of these patients, including age, mechanism of injury, operative diagnosis, type of surgical procedure, ICU treatment, and outcome, are presented in Table 4. All surgically treated children survived and were ultimately discharged home. Thirteen were admitted to the surgical ICU, while four were transferred directly to the neurosurgical ward after surgery without ICU admission. Operative diagnoses and procedures were extracted from the operative records.

The discharge categories “improved” and “recovered” in Table 4 were extracted from the discharge summaries and verified against the source documentation. They reflect the status recorded by the treating clinicians and were not based on a standardized neurological or functional outcome scale. Discharge destination is a separate variable; all 17 surgically treated children were discharged home.

**Table 4:** Clinical Characteristics, Surgical Procedures, and Outcomes of Surgically Treated Children (*n* = 17).

No.	Age	Mechanism of Injury	Operative Diagnosis	Type of Surgery	ICU Admission (YES/NO)	Discharge Status
1	37–72 months	Traffic accident	Skull and frontal skull-base fracture on the left. Depressed fracture of the superior wall of the left orbit. Dural displacement, cerebral laceration and contusion in the left frontobasal region. Lacerocontused wound of the frontal region. CSF leak. Pneumocephalus.	Bilateral frontal osteoplastic craniotomy. Removal of depressed bone fragments from the superior wall of the left orbit, debridement of the cerebral wound, suturing and duraplasty with periosteum. Primary suturing of the lacerocontused frontal wound.	YES	Improved
2	≤12 months	Fall from standing level	Epidural hematoma.	Left temporoparietal osteoplastic craniotomy. Evacuation of the epidural hematoma. Epidural space drainage.	YES	Improved
3	≤12 months	Cesarean section	Depressed calvarial fracture of the right parietal bone. Right parietal epidural hematoma.	Exploration. Simple trepanation of the right parietal bone. Craniotomy. Elevation of the bone flap. Irrigation and drainage. Repositioning of the bone flap. Evacuation of the epicranial hematoma. Drainage No. 1. Soft-tissue suturing.	YES	Improved
4	≤12 months	Fall from a height of 80 cm onto laminate flooring	Depressed temporoparietal skull fracture on the left.	Left parietal osteoclastic craniotomy. Elevation and leveling of the depressed bone.	YES	Recovered
5	37–72 months	Fall from a slide	Status post right temporal skull fracture. Right temporobasal epidural hematoma. Left hemiparesis.	Right-sided trauma flap. Evacuation of the right temporobasal epidural hematoma.	YES	Improved
6	13–36 months	Fall, unknown cause	Left temporoparietal skull fracture. Left temporoparietal epidural hematoma.	Left frontotemporoparietal osteoplastic craniotomy. Evacuation of the epidural hematoma. Subgaleal space drainage.	YES	Improved
7	37–72 months	A cabinet fell on the child	Skull-base fracture and multifragmentary depressed fracture of the right parietal region. Right intracerebellar hematoma. Left-sided otorrhea of cerebrospinal fluid. Right zygomatic bone fracture. Lacerocontused scalp wound on the right.	Right parietal osteoplastic craniotomy. Elevation and leveling of the depressed bone.	YES	Improved
8	37–72 months	A metal fence fell on the child	Right parietal epidural hematoma. Right parietal intracerebral hematoma. Traumatic subarachnoid hemorrhage. Multifragmentary right parietal skull fracture.	Left frontotemporoparietal osteoplastic craniotomy. Evacuation of epidural and intracerebral hematomas. Cerebral debridement. Duraplasty with periosteum. Subgaleal space drainage.	YES	Improved
9	≤12 months	Cesarean section	Depressed left temporoparietal skull fracture.	Elevation of a complex skull fracture.	YES	Improved
10	≤12 months	Delivery	Depressed fracture of the left parietal bone.	Osteoclastic craniectomy. Elevation of the depressed portion of the left parietal bone.	NO	Recovered
11	37–72 months	Horse kick	Multifragmentary depressed fracture of the right parietal region of the skull.	Other procedures on the maxillary and frontal sinus (elevation, fracture, fistula, etc.).	NO	Improved
12	≤12 months	Fall, unknown cause	Depressed left parietal skull fracture.	Simple trepanation. Elevation and leveling of the fragments.	NO	Recovered
13	≤12 months	Fall from a high chair	Left parietal calvarial fracture.	Simple trepanation. Elevation and leveling of depressed calvarial bone fragments.	NO	Improved
14	37–72 months	Traffic accident	Frontal bone fracture. Intracerebral hemorrhage, unspecified.	Elevation of a complex skull fracture.	YES	Improved
15	37–72 months	Fall, unknown cause	Right parietal skull fracture. Lacerocontused scalp wound in the right parietal region.	Wound exploration. Suturing.	YES	Recovered
16	37–72 months	Traffic accident	Frontal bone fracture.	Other procedures on the maxillary and frontal sinus (elevation, fracture, fistula, etc.).	YES	Improved
17	13–36 months	Fall down the stairs	Right parietal calvarial fracture.	Evacuation of the epidural hematoma.	YES	Recovered

Patients requiring intensive care were subsequently transferred to the Neurosurgery Department. All 17 surgically treated patients were ultimately discharged home.

### 3.6. Logistic Regression Model

A multivariable logistic regression model was fitted with severe clinical course as the binary outcome variable (0 = no, 1 = yes). Five categorical predictors were included: sex (male, female), age group (≤12 months, 13–36 months, 37–72 months), place of residence (rural, urban), mode of arrival (brought by parents, ambulance transport, transfer from another healthcare facility), and injury mechanism (falls, direct blow/struck/crush, traffic-related injury, other/unknown).

The composite indicator of severe clinical course was coded as positive if at least one of the following criteria was present: recorded GCS ≤ 8 on admission, treatment in the surgical intensive care unit, and/or surgical intervention during hospitalization. Overall, 70 children (7.2%) met the criteria for severe clinical course, while 901 (92.8%) did not. Six children had a recorded GCS ≤ 8, and all six were also treated in the surgical intensive care unit; therefore, the GCS criterion did not identify any additional severe cases beyond those identified by surgical intensive care treatment and/or surgical intervention. No evidence of problematic multicollinearity or overdispersion was detected in the fitted model. Complete or quasi-complete separation was assessed and was not detected. Results of the logistic regression—adjusted odds ratios, 95% confidence intervals, and *p*-values—are reported in Table 5.

Sex and place of residence were not statistically significantly associated with severe clinical course after adjustment.

Children aged 13–36 months had statistically significantly lower odds of a severe clinical course compared with children aged ≤12 months, adjusting for the other variables in the model. Children aged 37–72 months similarly had 71% lower odds of a severe clinical course than those aged ≤12 months. Overall, these results indicate that children aged ≤12 months had the highest odds of a severe clinical course among the age groups.

Mode of arrival was statistically significantly associated with a severe clinical course. Children transported by ambulance had more than four times the odds of severe clinical course compared with children brought by parents, adjusting for sex, age group, place of residence, and injury mechanism. Children transferred from another healthcare facility had more than six times the odds of severe clinical course compared with children brought by parents.

The injury mechanism was also statistically significantly associated with a severe clinical course. Compared with falls, direct blow/struck/crush injuries were associated with almost 2.5 times the odds of severe clinical course. Traffic-related injuries were associated with more than four times the odds of severe clinical course compared with falls, representing the strongest mechanism-related association. The heterogeneous other/unknown mechanism group was also associated with higher odds (2.82 times the odds) of severe clinical course compared with falls; however, this finding should be interpreted cautiously because the category combines different and unclear mechanisms.

Among the variables included in the model, interfacility transfer showed the largest adjusted association with severe clinical course (aOR = 6.54, 95% CI 3.35–12.78), followed by traffic-related injury (aOR = 4.33, 95% CI 1.98–9.49) and ambulance transport (aOR = 4.30, 95% CI 2.11–8.76).

The model showed modest explanatory ability, with Tjur’s R^2^ = 0.141 and good discrimination (5-fold cross-validated AUC = 0.811, 95% CI [0.771, 0.869]). Graphical assessment of calibration showed generally reasonable agreement between predicted and observed probabilities. The Hosmer–Lemeshow test showed no statistically significant evidence of lack of fit, χ^2^(8) = 11.37, *p* = 0.182.

As sensitivity analyses, the primary model was re-estimated using Firth penalized logistic regression and with calendar year included as an additional covariate. The Firth estimates were materially unchanged from those of the conventional logistic regression, with the same pattern of statistically significant and nonsignificant associations. Adjustment for calendar year also did not materially alter the estimates, and calendar year was not statistically significantly associated with severe clinical course in either the conventional logistic model (*p* = 0.920) or the Firth model (*p* = 0.930).

To examine whether the limited availability of GCS affected the primary analysis, the multivariable logistic regression was repeated using a severe-clinical-course outcome defined only by treatment in the surgical intensive care unit and/or surgical intervention. Excluding GCS did not alter the classification of any participant: the same 70 children were classified as having a severe clinical course and the same 901 as not having a severe clinical course. Consequently, the regression estimates were identical to those of the primary model.

As a more restrictive sensitivity analysis, surgical intervention alone was used as the outcome. Because only 17 children (1.8%) underwent surgery, Firth penalized logistic regression was used. The overall model was statistically significant, likelihood-ratio χ^2^(9) = 35.11, *p* < 0.001. Compared with children aged ≤12 months, children aged 13–36 months had lower odds of surgical intervention (aOR = 0.12, 95% CI [0.02, 0.48], *p* = 0.002), as did children aged 37–72 months (aOR = 0.27, 95% CI [0.09, 0.79], *p* = 0.017). Transfer from another healthcare facility was associated with higher odds of surgical intervention (aOR = 4.81, 95% CI [1.50, 15.78], *p* = 0.009), as were direct blow/struck/crush injuries (aOR = 4.63, 95% CI [1.04, 17.90], *p* = 0.045) and other/unknown injury mechanisms (aOR = 7.39, 95% CI [2.21, 24.71], *p* = 0.002). Ambulance transport showed a positive but nonsignificant association (aOR = 3.96, 95% CI [0.97, 14.61], *p* = 0.055), whereas traffic-related injury, sex, and place of residence were not significantly associated with surgical intervention. Given the small number of surgical events, these results should be interpreted cautiously.

## 4. Discussion

This hospital-based cohort confirms that falls predominate among hospitalized young children with head injury and that severe clinical courses are uncommon. The most distinctive finding was temporal: although the annual number of head-injury examinations decreased, the proportion hospitalized increased markedly and nonlinearly, reaching 58.0% in 2025. Because patient-level information was unavailable for non-hospitalized children, this trend cannot be attributed to changes in injury severity or case mix. Institutional changes, observation practices, referral patterns, diagnostic practice, coding, and other healthcare-system factors are plausible explanations, but none was directly tested in the present analysis. The hospitalization proportion should therefore be interpreted as a measure of service use rather than a surrogate for injury severity. This distinction is important when comparing hospitalization patterns across centers or over time [8,13,14].

Falls from heights within the home were the most frequent mechanism, consistent with previous pediatric head-injury studies [7,15,16]. From a prevention perspective, this finding reinforces the relevance of age-appropriate household safety measures and caregiver education, particularly around furniture, stairs, high chairs, and carrying infants. The seasonal concentration in summer and autumn may reflect greater activity and exposure, but the present study did not measure activity patterns and this interpretation remains descriptive.

GCS was documented in only 9.2% of hospitalizations, limiting its use as a stand-alone severity measure and underscoring an important limitation of retrospective routine data. Importantly, all six children with recorded GCS ≤ 8 were already captured by intensive-care admission, so removing GCS from the composite outcome did not change classification. Most children were managed conservatively, while intensive-care treatment and surgery were uncommon. Skull radiography was used much more often than CT in the cohort. Because imaging use was not analyzed by calendar year or age, the present data should not be used to infer temporal changes in imaging practice. NICE guidance provides a framework for head-injury assessment, observation, and imaging decisions [17]. Nevertheless, the overall predominance of skull radiography differs from current recommendations favoring validated clinical decision rules, observation, and selective CT rather than routine skull radiography [18,19,20,21].

Within the hospitalized cohort, age ≤ 12 months, ambulance transport, interfacility transfer, and several non-fall injury mechanisms were associated with a severe clinical course after adjustment. The associations with ambulance transport and transfer should not be interpreted as causal risk factors; they likely reflect triage and referral of children who appeared more concerning at initial assessment. Traffic-related injury was also associated with greater odds of a severe clinical course, consistent with the higher-energy nature of this mechanism [4,5,7]. Clinically, these findings support heightened vigilance when infants or children with high-energy mechanisms arrive through emergency transport or referral pathways, while emphasizing that mode of arrival itself is a marker of prehospital assessment rather than a determinant of outcome. In the surgery-only sensitivity analysis, the associations with ambulance transport and traffic-related injury were no longer statistically significant, and all surgery-only estimates require caution because only 17 surgical events occurred.

This study has several limitations. Its retrospective, single-center design limits generalizability. Case ascertainment relied on three primary ICD-10 discharge codes, so children with other intracranial injuries, skull fractures, or head injuries coded under another primary diagnosis may have been missed; coding heterogeneity over the long study period may also have affected ascertainment. GCS was undocumented in 90.8% of cases and the missingness should not be assumed to be random. The composite severe-course outcome includes intensive-care admission, which can be influenced by local practice and resource availability; the surgery-only sensitivity analysis partly addresses this issue but was limited by only 17 events. In addition, only aggregate annual counts were available for non-hospitalized head-injury presentations. We therefore could not compare hospitalized and non-hospitalized children at the patient level, evaluate changes in case mix or severity among all presentations, or identify determinants of admission. Population-based incidence and prevalence could not be estimated because the study did not include a defined population denominator or complete ascertainment of all head injuries in the catchment population. Finally, organizational, referral, imaging, coding, and COVID-era changes were not formally analyzed, so they remain possible rather than established explanations for the observed hospitalization trend.

The contribution of this study is the combination of a 16-year description of hospitalized young children in a Serbian tertiary center, analysis of severe clinical course within the admitted cohort, and formal analysis of annual hospitalization proportions. The observed mechanisms and age-related associations largely confirm existing evidence. The more distinctive observation is the divergence between declining head-injury examinations and rising hospitalization proportions. This is a local service-use signal warranting investigation rather than evidence of worsening population-level injury severity or inappropriate admission.

### Implications for Clinical Practice

The practical value of these findings is to identify measurable priorities for local quality improvement. The rising hospitalization proportion warrants periodic audit of documented admission indications, referral pathways, and observation capacity. The high proportion of undocumented GCS scores supports standardized, age-appropriate neurological assessment on arrival and repeated documentation during observation. Local pathways should specify observation, escalation, and discharge criteria consistent with established pediatric guidance [17]. Imaging practices should be reviewed against current pediatric recommendations [18,19,20,21], while imaging decision rules should be used for their validated purpose rather than as substitutes for admission criteria. Infants and children arriving through emergency referral pathways require careful clinical assessment, but age or mode of arrival alone should not determine admission. Household-fall prevention and targeted caregiver education remain relevant accompanying priorities. Implementation should be evaluated using both service-use and safety outcomes, including deterioration, unplanned return visits, and delayed recognition of clinically important injury. These proposals are quality-improvement priorities, not interventions shown to be effective by this study; the current cohort cannot identify which admissions could safely be avoided.

Future multicenter prospective studies should collect patient-level data for both hospitalized and non-hospitalized children, use broader and standardized case ascertainment, and measure changes in referral pathways, observation capacity, imaging, staffing, and clinical decision-rule implementation. Such data would allow investigators to distinguish changes in injury epidemiology from changes in healthcare delivery and to evaluate whether standardized observation pathways can reduce avoidable admissions while maintaining safety.

## 5. Conclusions

The central finding was a marked, nonlinear increase in the proportion hospitalized despite fewer annual head-injury examinations at this Serbian tertiary pediatric center. Available data cannot determine whether this reflects changes in case mix, referral pathways, coding, or organization of care. Within the hospitalized cohort, most children were managed conservatively, home falls predominated, and infancy, emergency transport or transfer, and selected mechanisms were associated with severe clinical course. The findings support audit of admission and observation pathways, improved neurological documentation, and prevention of domestic falls; they do not establish which admissions could safely be avoided.

## Figures and Tables

**Table 1:** Demographic Characteristics of the Sample (*N* = 971).

Characteristic	Category	*n* (%)
Sex	Male	519 (53.5%)
	Female	452 (46.5%)
Age	≤12 months	181 (18.6%)
	13–36 months	356 (36.7%)
	37–72 months	434 (44.7%)
Place of Residence	Urban	543 (55.9%)
	Rural	428 (44.1%)
Season	Winter	165 (17%)
	Spring	229 (23.6%)
	Summer	298 (30.7%)
	Fall	279 (28.7%)

**Table 2:** Mechanism of Injury.

Mechanism of Injury	*n* (%)
Fall from height—inside the home (bed, chair, caregiver’s arms, stairs, high chair, etc.)	378 (38.9%)
Fall on the same level (flat surface, no height, accidental fall)	166 (17.1%)
Blow or collision during play (ball, toy, horse, hit against a door)	93 (9.6%)
Fall from a vehicle or toy (bicycle, swing, carousel, tractor, stroller)	86 (8.9%)
Fall from height—outside the home (ladder, terrace, construction site/building)	73 (7.5%)
Unknown cause	73 (7.5%)
Traffic accident	61 (6.3%)
Object fell on the child inside the home (wardrobe, TV)	19 (2%)
Object fell on the child outside the home (branch, pole)	12 (1.2%)
Birth trauma	5 (0.5%)
Abuse	5 (0.5%)

**Table 3:** Clinical Characteristics of Injuries and Treatment.

Clinical Characteristic	Category	*n* (%)
Diagnosis	Concussion	465 (47.9%)
	Skull-vault fracture	205 (21.1%)
	Superficial head injury	301 (31.0%)
Symptoms	Neurological	678 (69.8%)
Symptoms	Somatic	195 (20.1%)
Symptoms	Psychological/behavioral	26 (2.7%)
Symptoms	Unreliable data	10 (1%)
Symptoms	Nonspecific	62 (6.4%)
Diagnostics	CT only	197 (20.3%)
Diagnostics	Skull radiography only	659 (67.9%)
Diagnostics	None	44 (4.5%)
Diagnostics	CT + Skull radiography	71 (7.3%)
Length of hospitalization	1 day	406 (41.8%)
Length of hospitalization	>1 day	565 (58.2%)
Department	Surgical intensive care	66 (6.8%)
Department	Ward	905 (93.2%)
Type of treatment	Conservative	954 (98.2%)
Type of treatment	Surgical	17 (1.8%)

**Table 5:** Multivariable Logistic Regression Model for Severe Clinical Course (*N* = 971).

Predictors	Category	Severe Clinical Course *n*/*N* (%)	aOR	95% CI	*p*
Sex	Male	31/519 (6.0)	ref.		
	Female	39/452 (8.6)	1.37	0.80–2.35	0.245
Age	≤12 months	27/181 (14.9)	ref.		
	13–36 months	18/356 (5.1)	0.30	0.15–0.60	<0.001
	37–72 months	25/434 (5.8)	0.29	0.15–0.55	<0.001
Place of residence	Rural	44/428 (10.3)	ref.		
	Urban	26/543 (4.8)	0.64	0.37–1.12	0.122
Arrival	Brought by parents	21/701 (3.0)	ref.		
	Ambulance transport	23/150 (15.3)	4.30	2.11–8.76	<0.001
	Transfer	26/120 (21.7)	6.54	3.35–12.78	<0.001
Injury mechanism	Falls	31/703 (4.4)	ref.		
	Direct blow/struck/crush	10/124 (8.1)	2.45	1.12–5.36	0.025
	Traffic-related	17/61 (27.9)	4.33	1.98–9.49	<0.001
	Other/unknown	12/83 (14.5)	2.82	1.30–6.13	0.009

Note. aOR = adjusted odds ratio. *n* = number of severe clinical course events within the category; *N* = total number of patients in the category. CI = confidence interval. Reference categories were male sex, age ≤ 12 months, rural residence, brought by parents, and falls. The outcome was severe clinical course, defined as recorded GCS ≤ 8 on admission, surgical ICU admission, and/or surgical intervention during hospitalization. Transfer = transfer from another healthcare facility.

## Data Availability

The data that support the findings of this study are available on request from the corresponding author.

## Abbreviations

THI	Traumatic head injury
GCS	Glasgow Coma Scale
CT	Computed tomography
